# Abnormal brain spontaneous neural activity in neuromyelitis optica spectrum disorder with neuropathic pain

**DOI:** 10.3389/fneur.2024.1408759

**Published:** 2024-06-12

**Authors:** Gendi Wang, Xiang Chen, Xiaoyuan Wang, Yinghui Duan, Hanqing Gao, Xiaopei Ji, Yunfei Zhu, Xuanyi Xiang, Hairong Ma, Yonggang Li, Qun Xue

**Affiliations:** ^1^Department of Neurology, The First Affiliated Hospital of Soochow University, Suzhou, China; ^2^Department of Neurology, Yancheng Third People’s Hospital, Yancheng, China; ^3^Department of Radiology, The First Affiliated Hospital of Soochow University, Suzhou, China; ^4^Department of Neurology, Kunshan Hospital of Chinese Medicine, Suzhou, China; ^5^Institute of Medical Imaging, Soochow University, Suzhou, China; ^6^National Clinical Research Center for Hematologic Diseases, The First Affiliated Hospital of Soochow University, Suzhou, China; ^7^Medical College of Soochow University, Suzhou, China; ^8^Jiangsu Key Laboratory of Clinical Immunology, Jiangsu Institute of Clinical Immunology, The First Affiliated Hospital of Soochow University, Suzhou, China

**Keywords:** neuromyelitis optica spectrum disorder, neuropathic pain, amplitude of low-frequency fluctuations, anterior insula, amygdala

## Abstract

**Background:**

Neuropathic pain is one of the most common symptoms in neuromyelitis optica spectrum disorder (NMOSD). Notwithstanding, its underlying mechanism remains obscure.

**Methods:**

The amplitude of low-frequency fluctuations (ALFF) metric was employed to investigate spontaneous neural activity alterations via resting-state functional magnetic resonance imaging (rs-MRI) data from a 3.0 T MRI scanner, in a sample of 26 patients diagnosed with NMOSD with neuropathic pain (NMOSD-WNP), 20 patients with NMOSD but without neuropathic pain (NMOSD-WoNP), and 38 healthy control (HC) subjects matched for age and sex without the comorbidity of depressive or anxious symptoms.

**Results:**

It was observed that patients with NMOSD-WNP displayed a significant ALFF decrease in the left amygdala and right anterior insula, relative to both patients with NMOSD-WoNP and HC subjects. Furthermore, ALFF values in the left amygdala were negatively correlated with the scores of the Douleur Neuropathique en 4 Questions and McGill Pain Questionnaire (both sensory and affective descriptors) in patients with NMOSD-WNP. Additionally, there were negative correlations between the ALFF values in the right anterior insula and the duration of pain and the number of relapses in patients with NMOSD-WNP.

**Conclusion:**

The present study characterizes spontaneous neural activity changes in brain regions associated with sensory and affective processing of pain and its modulation, which underscore the central aspects in patients with NMOSD-WNP. These findings might contribute to a better understanding of the pathophysiologic basis of neuropathic pain in NMOSD.

## Introduction

Neuromyelitis optica spectrum disorder (NMOSD) is an inflammatory disorder affecting the central nervous system, predominantly the optic nerves and spinal cord, resulting in a range of symptoms such as vision loss, muscle weakness, and pain (1). In China, NMOSD is seen as one of the most common types of the central nervous system (CNS) inflammatory demyelinating diseases, with an estimated incidence of 0.278 per 100,000 individuals (2, 3). Pain, especially the neuropathic character, can be detected in more than 80% of NMOSD patients (4). Management of neuropathic pain is still a challenge, diminishing the health-related quality of life of patients and increasing the economic burden of individuals (5–7). These facts point to the necessity of gaining a more thorough understanding of the neurobiological basis of NMOSD associated with neuropathic pain, so as to improve treatment strategies.

In the last two decades, noninvasive MRI techniques have been a critical component for understanding brain reorganization in NMOSD. By employing a range of structural and functional MRI (fMRI) techniques and analytic metrics, significant changes in brain gray matter volume (8–11), cortical thickness (12), white matter volume (10, 13, 14), white matter microstructure (15), resting-state regional homogeneity (16), spontaneous neuronal activity (17), cerebral blood flow (18), neurovascular coupling (19), functional connectivity (11, 20–22), and structural and functional networks (21, 23–29) have been observed in NMOSD. These changes have been linked to visual impairment, cognitive dysfunction, fatigue, disease severity, disease duration, and other clinical features in NMOSD. These studies have increased knowledge of the pathophysiological mechanisms of NMOSD.

So far, only a few structural MRI studies have been conducted to investigate brain reorganization in NMOSD patients with neuropathic pain (MNOSD-WNP) (30–32). A structural MRI study demonstrated that patients with neuromyelitis optica (NMO) and neuropathic pain had reduced volumes of the hippocampus and pallidum. Additionally, a significant negative correlation was observed between the Brief Pain Inventory score and the volumes of the accumbens nucleus and thalamus in those with NMO (31). Asseyer et al. (32) found an inverse correlation of the ventral posterior nucleus of the thalamus with neuropathic pain intensity in patients with NMOSD. Results from another study indicated that neuropathic pain in NMOSD was linked to a decrease in the local gyrification index of the left temporal lobe and its surrounding areas, as well as alterations in the white matter skeleton of the corticospinal tract and thalamocortical tract (30). Research has shown that neuropathic pain in conditions such as trigeminal neuralgia, postherpetic neuralgia, and spinal cord injury is linked to changes in regional spontaneous neural activity as evidenced by the amplitude of low-frequency fluctuations (ALFF) metric of rest-state fMRI, which is involved in pain perception and its modulation (33–35). ALFF is a widely used approach to measure the low-frequency (0.01–0.1 Hz) fluctuations of the brain blood oxygen level dependent (BOLD) signal, which is an indicator of regional neural activity during rest (36). As yet, no studies have been conducted to investigate regional brain activity changes in patients with NMOSD suffering from neuropathic pain.

This study sought to gain an initial insight into the neuropathophysiological basis of neuropathic pain in NMOSD through the utilization of the resting-state ALFF approach. We examined ALFF differences among patients with NMOSD-WNP, NMOSD patients without neuropathic pain (NMOSD-WoNP), and healthy control (HC) subjects. Examining the associations between regional brain activity, pain rating scales, and pain duration was also conducted.

## Methods

### Participants

A total of 46 patients with NMOSD were consecutively recruited into the study from the Department of Neurology of the First Affiliated Hospital of Soochow University from January 2021 to November 2021. Patients were enrolled in the study if they (1) were diagnosed with NMOSD in accordance with the 2015 International Panel Consensus Diagnostic criteria (37); (2) were evaluated during the remission phase without taking high-dose steroids at least 4 weeks before the MRI examination; (3) were aged 18 to 70 years and right-handed; and (4) had no anxious and depressive symptoms: Hamilton Anxiety Rating Scale (HAMA) score <7 and Hamilton Depression Rating Scale (HAMD-17) score <7. In order to screen for neuropathic pain in patients with NMOSD, the Douleur Neuropathique en 4 Questions (DN4) assessment tool was utilized. A score of DN4 ≥4/10 is indicative of neuropathic pain (38). The patients with NMOSD-WNP were further subjected to a Chinese form of the McGill Pain Questionnaire (MPQ), which featured a core set of 12 sensory descriptors and an ancillary set of 4 affective descriptors. The present extent of pain severity was assessed using a visual analog scale (VAS). Of these patients, 26 were diagnosed with NMOSD-WNP and 20 were diagnosed with NMOSD-WoNP.

Thirty-eight age- and sex-matched right-handed healthy controls (HCs) from the local community were recruited by advertisement. The exclusion of participants was based on the following criteria: (1) a contraindication for the MRI scan; (2) a history of head trauma, psychiatric disorders, and neurological or pain-related disorders other than NMOSD-WNP and NMOSD-WoNP; (3) visible brain lesions on conventional MRI; and (4) substance abuse.

To determine the degree of disability of patients with NMOSD, the Expanded Disability Status Scale (EDSS) was utilized. The severity of anxiety and depressive symptoms was evaluated using HAMA and HAMD-17, respectively. Serum anti-aquaporin-4 autoantibodies (AQP4-Ab) were detected by enzyme linked immunosorbent assay.

The study received approval from the local ethical standards committee (code number: 007/2020), and all participants granted written informed consent prior to participation.

### MRI acquisition

Imaging data were obtained from a 3.0 T MRI scanner (Philips Ingenia, Philips Healthcare, Best, The Netherlands). All the participants were told to stay still, keep their eyes closed, stay awake, and think of nothing during the scanning process. Data for rs-fMRI was obtained through the use of gradient-echo echo-planar imaging (EPI) sequence, which included a TR of 2,000 ms, a TE of 30 ms, a FA of 90°, a FOV of 240 × 240 mm^2^, a slice thickness of 4 mm, a slice gap of 0.4 mm, 30 interleaved axial slices, and 250 volumes for each subject.

### Imaging data preprocessing

Preprocessing of the resting state fMRI data was carried out using DPABI 4.3^1^ (39) and SPM12^2^ software packages on the Matlab R2020b platform. This included quality assessment of all subjects’ images, conversion of the DICOM format of the rs-fMRI raw data to the NIFTI format, removal of the first 10 scan volumes to ensure steady-state magnetization and signal stabilization, slice timing correction to eliminate differences in imaging acquisition time, realignment to correct head motion, spatial normalization to the Montreal Neurological Institute (MNI) template with a resampling voxel size of 3 × 3 × 3 mm^3^, spatial smoothing with a 6 × 6 × 6 mm^3^ Gaussian kernel box, removal of linear trend, regression of covariates such as head motion using the Friston 24 parameter, global mean signal, white matter signal and cerebrospinal fluid signal, and band-pass filtering to reduce the effects of high-frequency noise and low-frequency drifts (0.01–0.1 Hz). To reduce the influence of micro head movements on the results, we chose participants with a low mean framewise displacement (FD) as calculated using Jenkinson’s formula (mean FD <0.2 mm). Owing to excessive head movements, two patients and one control subject were removed from the study, with their mean FD surpassing 0.2 mm. The preprocessed fMRI data was then utilized for further analysis.

### ALFF analysis

ALFF analysis was performed utilizing the DPABI software (39). The ALFF value was calculated through the following steps: the time series of each voxel, ranging from 0.01 to 0.1 Hz, was transformed into the average value of the square root by the fast Fourier transform algorithm, which was calculated as the ALFF. To optimize statistical analysis, ALFF maps were additionally smoothed with an 8 mm full width at half maximum Gaussian kernel. The voxels were then normalised by the average ALFF value of each participant, within the default brain mask of the DPABI tool box. This approach produced a standardized brain ALFF map.

### Statistical analysis

Statistical analyses were conducted on demographic and clinical data using the SPSS software (version 27.0, Chicago, IL, United States). One-way analysis of variance (ANOVA) was used to compare the age, years of education, HAMD-17 score, and HAMA score among the three groups. The differences in illness duration, the number of relapses, and EDSS scores between the NMOSD-WNP and NMOSD-WoNP groups were assessed by independent two-sample *t*-tests. The categorical variables of gender distribution and AQP4-Ab status were subjected to Fisher’s exact and chi-square tests, respectively. The Bonferroni correction was implemented in the *post hoc* comparisons. The threshold for statistical significance was set at a *p*-value of less than 0.05.

To find the main ALFF differences among the three groups, a one-way analysis of covariance (ANCOVA) model was conducted in a voxel-wise manner with age, sex, education level, HAMD-17 score, and HAMA score as nuisance covariates. The corrected value for ANCOVA was set at *p* < 0.05 using the family wise error (FWE) correction with a permutation-based approach with threshold-free cluster enhancement (TFCE) method. Subsequently, the clusters with significant differences in ALFF among the three groups were selected as the regions of interest (ROIs). The mean ALFF *z*-values of these clusters were extracted for every subject and compared between each pair of the three groups with Bonferroni correction (*p* < 0.05). Additionally, partial correlation analyses were made to investigate the relationships between the ALFF values in the ROIs and the pain duration, VAS, DN4, and MPQ (sensory and affective descriptors) scores in patients with NMOSD-WNP. Statistical significance was set at the level of *p* < 0.05 after removing the depression and anxiety effects.

## Results

### Demographic and clinical characteristics

Table 1 summarizes the demographic and clinical characteristics of the 26 patients with NMOSD-WNP (25 females, mean age 46.19 ± 13.80 years), 20 patients with NMOSD-WoNP (15 females, mean age 39.80 ± 14.50 years), and 38 HC subjects (31 females, mean age 42.42 ± 13.89 years). The three groups had significantly different scores of the HAMD-17 (*F* = 15.69, *p* < 0.001) and HAMA (*F* = 9.21, *p* < 0.001). There were no significant differences in age (*F* = 1.23, *p* = 0.3), gender (*χ*^2^ = 2.97, *p* = 0.23), or education level (*F* = 1.3, *p* = 0.28) among the groups. Significant differences were observed regarding the illness duration (*p* = 0.032) and number of relapses (*p* = 0.039) between patients with NMOSD-WNP and patients with NMOSD-WoNP. There were no significant differences in EDSS (*p* = 0.091) or AQP4-Ab status (*p* = 0.88) between patients with NMOSD-WNP and patients with NMOSD-WoNP.

**Table 1:** Demographic and clinical features of all participants.

	NMOSD-WNP (*n* = 26)	NMOSD-WoNP (*n* = 20)	HC (*n* = 38)	Statistic	*p*-value		
					NMOSD-WNP vs. HC	NMOSD-WoNP vs. HC	NMOSD-WNP vs. NMOSD-WoNP
Age	46.19 ± 13.80	39.80 ± 14.50	42.42 ± 13.89	*F* = 1.23	0.29	0.5	0.13
Sex (M/F)	1:25	5:15	7:31	*χ^2^* = 2.97	0.08	0.74	0.18
Education (years)	9.65 ± 4.86	10.65 ± 4.99	11.76 ± 5.46	*F* = 1.302	0.113	0.438	0.519
Illness duration (years)	5.06 ± 5.73	2.10 ± 1.94	—	*T* = 2.21	—	—	0.032^*^
Number of relapses	3.04 ± 2.13	1.95 ± 0.94	—	*T* = 2.13	—	—	0.039^*^
AQP4-Ab positive/negative	19:7	15:5	—	*χ^2^* = 0.02	—	—	0.88
EDSS	2.63 ± 2.27	1.71 ± 1.28	—	*T* = 1.731	—	—	0.091
HAMD-17	4.15 ± 2.26	3.25 ± 2.07	1.32 ± 1.68	*F* = 17.27^*^	0.000^*^	0.002^*^	0.38
HAMA	3.96 ± 2.16	2.95 ± 1.90	2.16 ± 2.77	*F* = 6.66^*^	0.001^*^	0.30	0.37
Pain duration (years)	3.95 ± 4.79	—	—	—	—	—	—
VAS	5.12 ± 1.53	—	—	—	—	—	—
DN4	5.00 ± 1.02	—	—	—	—	—	—
MPQ_Affective	3.04 ± 2.14	—	—	—	—	—	—
MPQ_Sensory	5.35 ± 2.48	—	—	—	—	—	—

NMOSD-WNP, neuromyelitis optica spectrum disorder with neuropathic pain; NMOSD-WoNP, neuromyelitis optica spectrum disorder without neuropathic pain; HC, healthy control; AQP4-Ab, anti-aquaporin-4 autoantibodies; EDSS, Expanded Disability Status Scale; HAMD, Hamilton Depression Rating Scale; HAMA, Hamilton Anxiety Rating Scale; NA, not available; VAS, visual analog scale; DN4, Douleur Neuropathique en 4 Questions; MPQ, McGill Pain Questionnaire; ^*^p < 0.05.

### ALFF analysis

**Figure 1:**
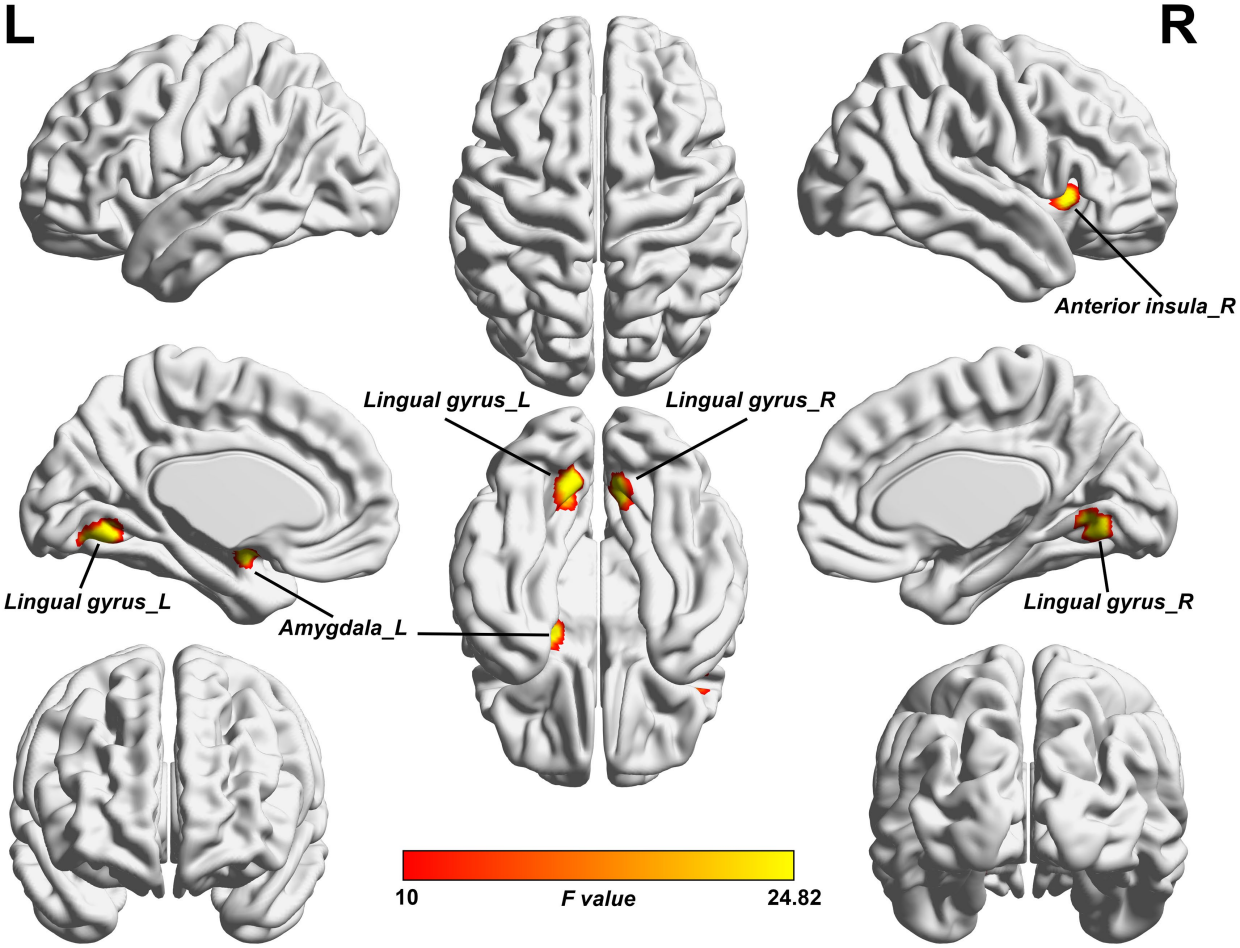
Clusters of significant ALFF differences among the NMOSD-WNP, NMOSD-WoNP, and HC groups. The *F*-map was created by ACONVA analysis of ALFF differences among the NMOSD-WNP, NMOSD-WoNP, and HC groups by family wise error (FWE) correction with a permutation-based approach with threshold-free cluster enhancement (TFCE) method (*p* < 0.05). ALFF, amplitude of low-frequency fluctuations; NMOSD-WNP, neuromyelitis optica spectrum disorder with neuropathic pain; NMOSD-WoNP, neuromyelitis optica spectrum disorder without neuropathic pain; HC, healthy control; R, right; L, left.

**Figure 2:**
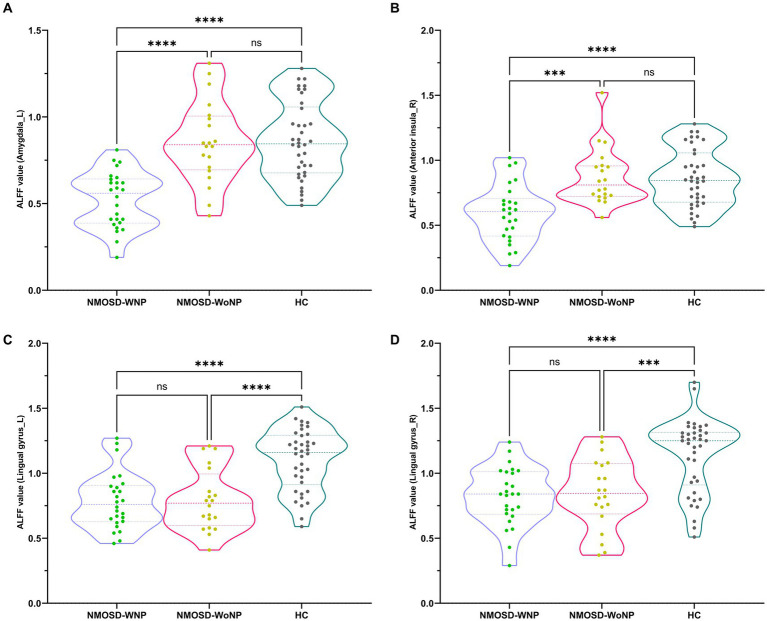
*Post hoc* comparisons of ALFF value differences at peak voxel between each pair group (NMOSD-WNP vs. HC, NMOSD-WoNP vs. HC, and NMOSD-WNP vs. NMOSD-WoNP) (Bonferroni correction). **(A–D)** denoted the ALFF differences in the left amygdala, right anterior insula, left lingual gyrus, and right lingual gyrus, respectively, across each pair group. ALFF, amplitude of low-frequency fluctuations; NMOSD-WNP, neuromyelitis optica spectrum disorder with neuropathic pain; NMOSD-WoNP, neuromyelitis optica spectrum disorder without neuropathic pain; HC, healthy control; R, right; L, left; ns, non-significant; ***p < 0.005; ****p < 0.001.

As shown in Figure 1 and Table 2, the ANCOVA test among the three groups revealed significant differences of the ALFF values in the left amygdala, right anterior insula, left lingual gyrus, and right lingual gyrus. Within these significant regions, *post hoc* analyses showed lower ALFF values in the left amygdala (Figure 2A) and right anterior insula (Figure 2B) in the NMOSD-WNP group relative to the NMOSD-WoNP group and the HC group. Relative to the HC group, the NMOSD-WNP and NMOSD-WoNP groups had lower ALFF values in bilateral lingual gyri (Figures 2C,D).

**Table 2:** Clusters showing significant ALFF differences among groups.

Brain regions	Peak MNI (*x*, *y*, *z*)	Cluster size (mm^3^)	Max *F* value	ALFF value (Mean with SD)			Post-hoc contrast results
				NMOSD-WNP	NMOSD-WoNP	HC	
Left amygdala	−22, 0, −16	112	24.82	0.52 ± 0.16	0.90 ± 0.24	0.91 ± 0.17	NMOSD-WNP < NMOSD-WoNP, NMOSD-WNP < HC, NMOSD-WoNP = HC
Right anterior insula	40, 20, 0	165	22.24	0.59 ± 0.22	0.87 ± 0.22	0.86 ± 0.22	NMOSD-WNP < NMOSD-WoNP, NMOSD-WNP < HC, NMOSD-WoNP = HC
Left lingual gyrus	−12, −68, −8	173	20.58	0.79 ± 0.22	0.79 ± 0.24	1.10 ± 0.23	NMOSD-WNP < HC, NMOSD-WONP < HC, NMOSD-WNP = NMOSD-WoNP
Right lingual gyrus	11, −65, −3	128	19.27	0.82 ± 0.22	0.84 ± 0.27	1.14 ± 0.28	NMOSD-WNP < HC, NMOSD-WONP < HC, NMOSD-WNP = NMOSD-WoNP

MNI, Montreal Neurological Institute; ALFF, amplitude of low-frequency fluctuations; SD, standard deviations; NMOSD-WNP, neuromyelitis optica spectrum disorder with neuropathic pain; NMOSD-WoNP, neuromyelitis optica spectrum disorder without neuropathic pain; HC, healthy control.

### Correlation analysis

**Figure 3:**
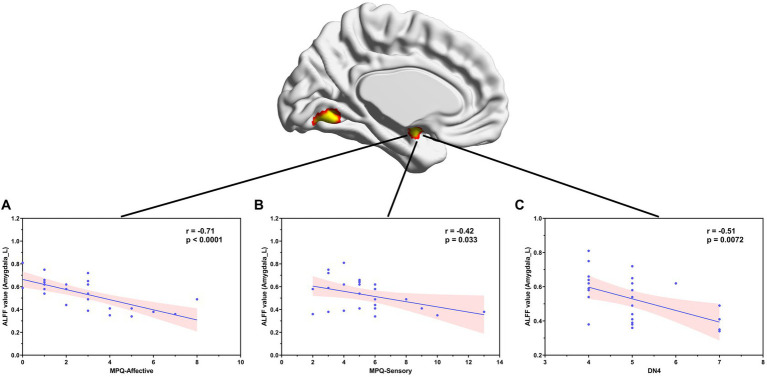
Correlation between ALFF values in the left amygdala and MPQ (both affective **(A)** and sensory **(B)** descriptors) and the DN4 scores **(C)** in patients with NMOSD-WNP. ALFF, amplitude of low-frequency fluctuations; NMOSD-WNP, neuromyelitis optica spectrum disorder with neuropathic pain; MPQ, McGill Pain Questionnaire; DN4, Douleur Neuropathique en 4 Questions; R, right; L, left.

**Figure 4:**
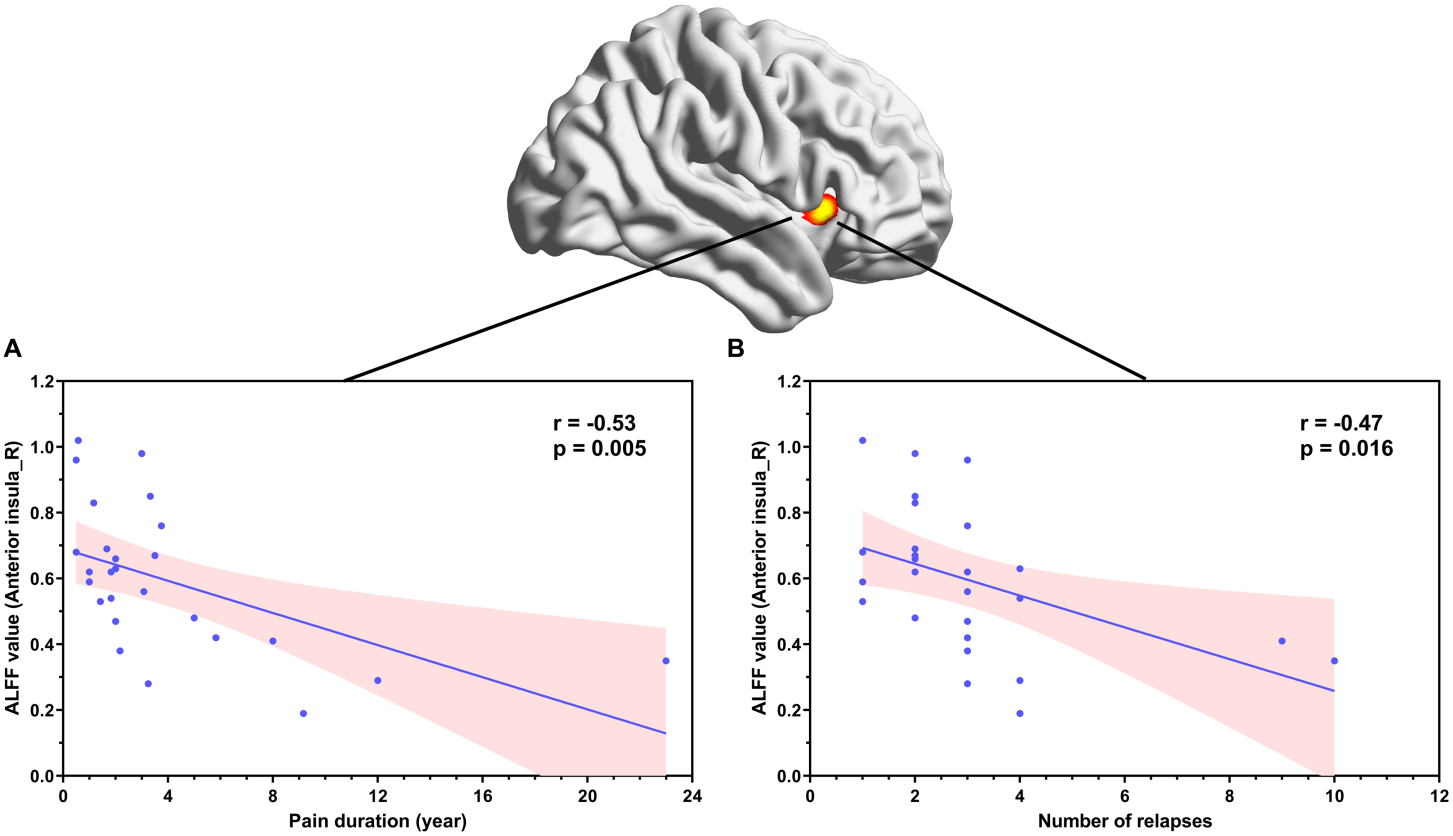
Correlation between ALFF values in the right anterior insula and pain duration **(A)** and number of relapses **(B)** in patients with NMOSD-WNP. ALFF, amplitude of low-frequency fluctuations; NMOSD-WNP, neuromyelitis optica spectrum disorder with neuropathic pain; R, right; L, left.

Correlation analyses showed that ALFF values in the left amygdala in patients with NMOSD-WNP were negatively correlated with the MPQ affective score (Figure 3A, *R* = −0.71, *p* < 0.001), MPQ sensory score (Figure 3B, *R* = −0.42, *p* = 0.033), and DN4 score (Figure 3C, *R* = −0.51, *p* = 0.0072). Pain duration and the number of relapses in the NMOSD-WNP were negatively correlated with the ALFF values in the right anterior insula (Figure 4A, *R* = −0.53, *p* = 0.005, and Figure 4B, *R* = −0.47, *p* = 0.016, respectively). No correlations were observed between the VAS and the ALFF values in the left amygdala or the right anterior insula.

## Discussion

To the best of our knowledge, this is the first research using the ALFF approach to explore the neural correlation of neuropathic pain in NMOSD. Our results demonstrated significantly decreased ALFF in the left amygdala and right anterior insula in patients with NMOSD-WNP compared to those with NMOSD-WoNP and HC subjects. Furthermore, our study uncovered that there were inverse correlations between the ALFF values in the left amygdala and the DN4 and MPQ scores (both sensory and affective descriptors), as well as negative correlations between the ALFF values in the right anterior insula and the pain duration and the number of relapses in patients with NMOSD-WNP.

One noteworthy finding of our study was that patients with NMOSD-WNP had decreased ALFF in the left amygdala in comparison to those with NMOSD-WoNP and HCs. The amygdala is a key part of the limbic system which plays a critical role in the emotional-affective element of pain and its modulation (40, 41). Animal studies have revealed that the amygdala, especially the central nucleus, is implicated in neuropathic pain behavior (42, 43). Structural MRI research has revealed a decrease in gray matter volume in the amygdala of individuals suffering from other neuropathic pain conditions, such as postherpetic neuralgia (44) and trigeminal neuralgia (45). An arterial spin labeling study showed increased cerebral blood flow in the amygdala in patients with postherpetic neuralgia (46). Another study utilizing rs-fMRI revealed a reduction in regional homogeneity in the amygdala in patients with idiopathic trigeminal neuralgia (47). Abnormal resting state amygdalar functional connectivity in the pain modulatory circuits was also observed in adolescents with neuropathic pain (40). In addition, research has shown that amygdalar functional connectivity was associated with both pain duration (45, 48) and emotional state ratings (45) in trigeminal neuralgia. Moreover, differences in pre-operative amygdalar functional connectivity between surgical responders and non-responders have been observed (48) and abnormal amygdalar functional connectivity could be adjusted following pain alleviation (45) in trigeminal neuralgia. Our research provided evidence of decreased spontaneous activity in the left amygdala in patients with NMOSD-WNP. Moreover, negative correlations between the ALFF values in the left amygdala and the DN4 and MPQ scores (both sensory and affective descriptors) were observed in patients with NMOSD-WNP, thus underscoring the importance of the amygdala in neuropathic pain. The finding of ALFF changes in the left amygdala may be particularly revealing to understand the neural basis of the neuropathic pain in NMOSD.

Convergent evidence suggests that the insula, which has reciprocal anatomical connections with multiple sensory, motor, limbic, and association areas and acts a component of the pain matrix, plays a critical role in the pathogenesis of chronic pain (49). Research suggests the posterior part of the insula is primarily engaged in the basic sensory aspects of pain, with the anterior insula cortex being more engaged in its affective/emotional and cognitive aspects (49). According to a meta-analysis of voxel-based morphometry studies, the gray matter volume in the bilateral anterior insula was found to be reduced in patients suffering from neuropathic pain conditions (50). Another meta-analysis of task-related fMRI studies revealed increased activation of the right caudal anterior insula in the presence of neuropathic pain compared to that observed in the presence of experimental pain (51). Studies have also demonstrated functional connectivity alterations of the anterior insula with other brain regions and networks in neuropathic pain conditions (52, 53). An animal model study further uncovered plasticity changes in functional activity and connectivity in the anterior insula, which were associated with the development of neuropathic pain (54). Our rs-fMRI investigation demonstrated decreased spontaneous activity in the anterior insula in patients with NMOSD-WNP, thereby corroborating the already existing evidence of its contribution to neuropathic pain. Moreover, our study revealed that ALFF values in the anterior insula were negatively correlated with pain duration and the number of relapses in patients with NMOSD-WNP. This observation indicates that the anterior insula may have a significant impact on the chronification of neuropathic pain in NMOSD.

There are several limitations of our study. Firstly, although we excluded participants with anxious and depressive symptoms from our study, significant differences were observed regarding the scores of HAMD-17 and HAMA among them. Nevertheless, patients’ characteristics, such the scores of HAMD-17 and HAMA, illness duration, and number of relapses were adjusted in the imaging analyses. Secondly, because of the cross-sectional nature of the study, whether brain ALFF abnormalities are altered by neuropathic pain remain unclear. Additional longitudinal research is thus required. Thirdly, our sample size was relatively small, which may have led to the failure of detecting certain differences among groups. Due to the limited sample size, we are also overlooking the possible impact of anti-AQP4 antibodies on brain function. In our study, patients with visible brain lesions on conventional MRI were excluded. Nevertheless, brain lesions/atrophy are frequently observed in NMOSD, with an increasing body of literature indicating a potential link to disease severity (55–57). The association between brain lesions/atrophy and neuralgia necessitates additional investigation. It is essential for forthcoming investigations to include larger sample sizes in order to confirm the validity of our results.

## Conclusion

We used the ALFF approach via rs-fMRI data to characterize spontaneous neural activity associated with neuropathic pain in NMOSD. Our study has yielded fresh evidence indicating decreased ALFF, primarily in the amygdala and anterior insula in patients with MNOSD-WNP. More specifically, the ALFF reduction in the amygdala correlated with neuropathic symptoms and signs that may be associated with the sensory and affective processing of pain. In contrast, the ALFF reduction in the right anterior insula may be associated with the chronification of neuropathic pain in patients with NMOSD. These findings suggest the existence of a distinct neural pattern that underlies the central aspects of neuropathic pain in MNOSD-WNP.

## Data availability statement

The raw data supporting the conclusions of this article will be made available by the authors, without undue reservation.

## Ethics statement

The studies involving humans were approved by the Ethics Committee of the First Affiliated Hospital of Soochow University. The studies were conducted in accordance with the local legislation and institutional requirements. The participants provided their written informed consent to participate in this study.

## Author contributions

GW: Writing – original draft, Data curation, Formal analysis, Project administration, Software. XC: Investigation, Methodology, Software, Visualization, Writing – original draft. XW: Data curation, Formal analysis, Methodology, Project administration, Software, Writing – review & editing. YD: Data curation, Formal analysis, Methodology, Writing – review & editing. HG: Data curation, Investigation, Project administration, Writing – review & editing. XJ: Data curation, Investigation, Writing – review & editing. YZ: Data curation, Investigation, Writing – review & editing. XX: Data curation, Investigation, Supervision, Writing – review & editing. HM: Conceptualization, Validation, Writing – review & editing. YL: Conceptualization, Validation, Writing – review & editing. QX: Conceptualization, Writing – review & editing, Funding acquisition, Validation.

## Footnotes

^1^ http://rfmri.org/DPABI

^2^ https://www.fil.ion.ucl.ac.uk/spm/
